# The Evolution of Nanoparticle Regulation: A Meta-Analysis of Research Trends and Historical Parallels (2015–2025)

**DOI:** 10.3390/nano16020134

**Published:** 2026-01-19

**Authors:** Sung-Kwang Shin, Niti Sharma, Seong Soo A. An, Meyoung-Kon (Jerry) Kim

**Affiliations:** 1 Department of Global Culture & Contents, Hankuk University of Foreign Studies, Dongdaemun-gu, Seoul 02450, Republic of Korea; sungkwangshin@outlook.com; 2 Bionano Research Institute, Gachon University, Seongnam-si 13120, Republic of Korea; nitisharma@gachon.ac.kr; 3 Department of Biochemistry & Molecular Biology, Korea University College of Medicine, Seongbuk-gu, Seoul 02841, Republic of Korea

**Keywords:** nanoparticles regulation, text mining analysis, topic modeling, TF-IDF, nanotechnology safety, interdisciplinary research

## Abstract

**Objective**: We analyzed nanoparticle regulation research to examine the evolution of regulatory frameworks, identify major thematic structures, and evaluate current challenges in the governance of rapidly advancing nanotechnologies. By drawing parallels with the historical development of radiation regulation, the study aimed to contextualize emerging regulatory strategies and derive lessons for future governance. **Methods**: A total of 9095 PubMed-indexed articles published between January 2015 and October 2025 were analyzed using text mining, keyword frequency analysis, and topic modeling. Preprocessed titles and abstracts were transformed into a TF-IDF (Term Frequency–Inverse Document Frequency) document–term matrix, and NMF (Non-negative Matrix Factorization) was applied to extract semantically coherent topics. Candidate topic numbers (K = 1–12) were evaluated using UMass coherence scores and qualitative interpretability criteria to determine the optimal topic structure. **Results**: Six major research topics were identified, spanning energy and sensor applications, metal oxide toxicity, antibacterial silver nanoparticles, cancer nano-therapy, and nanoparticle-enabled drug and mRNA delivery. Publication output increased markedly after 2019 with interdisciplinary journals driving much of the growth. Regulatory considerations were increasingly embedded within experimental and biomedical research, particularly in safety assessment and environmental impact analyses. **Conclusions:** Nanoparticle regulation matured into a dynamic multidisciplinary field. Regulatory efforts should prioritize adaptive, data-informed, and internationally harmonized frameworks that support innovation while ensuring human and environmental safety. These findings provide a data-driven overview of how regulatory thinking was evolved alongside scientific development and highlight areas where future governance efforts were most urgently needed.

## 1. Introduction

Nanoparticles became now to be widely used across industries, from pharmaceuticals and electronics to agriculture and environmental science [1]. In regulatory contexts, nanoparticles were commonly defined based on size, although definitions differ across jurisdictions. For example, the United States typically refered to materials in the flexible guidance, centered at the 1–100 nm range [2]. In contrast, Canada used a clearer working definition that covered materials within this size range or those that showed nanoscale properties, even if they were slightly larger [3]. The European Union (EU) applied a broader definition, based on the European Commission (EC) recommendation (2011/696/EU) that also considered particle number distributions and aggregation states [4]. Under the revised definition (2022/C 229/01), a nanomaterial was defined as a natural or manufactured materials of solid particles, either alone or clustered, in which at least 50% of the particles met specific nanoscale size or shape criteria [5]. Compared to the 2011 recommendation, the low surface area clause and the flexible 1–50% threshold were removed, leaving a fixed 50% cutoff [6]. Their increasing presence in consumer products revealed both promising applications and potential risks to health and the environment. As nanotechnology advances, regulatory infrastructure must evolve to manage the safe production, use, and disposal of nanoparticles [1]. These expanding applications and risks demanded inputs from multiple disciplines, including toxicology, materials science, environmental health, law, and ethics [7]. The rapid pace of nanotechnology development and the diverse nature of nanoparticles presented significant challenges in establishing and implementing comprehensive regulatory frameworks. Scientific uncertainties about environmental fate, bioaccumulation, and long-term effects of nanoparticles remained key barriers to evidence-based regulatory policies [8,9]. These uncertainties directly affected regulatory decision-making, particularly with respect to hazard classification, exposure assessment, and the applicability of existing chemical safety frameworks to nanoscale materials. As a result, experts from different fields needed to work together, and regulations must be updated regularly, as new research findings became available.

Historical regulatory precedents offered useful models for governing emerging technologies. The development of nanoparticle regulation resembled the evolution of radioactive material regulation in several ways [10,11]. While nanoparticles needed specific approaches due to their unique properties, the radiation regulation history provided relevant lessons. Radiation governance was especially relevant as a comparator, because it illustrated how regulatory systems evolved in response to scientific uncertainty, delayed evidence of harm, and the needs for international coordination and challenges that were increasingly evident in nanoparticle regulation. The field’s experience with risk assessment and international cooperation could inform nanoparticle governance [12,13], showing how scientific communities progressed from discovery to comprehensive safety protocols. These applied lessons to nanotechnology might help in developing better practical guidelines on nanoparticle classifications, setting safe limits for exposure, and monitoring their environmental effect.

Nanoparticle research progressed quickly from the pioneered theoretical concepts by Richard Feynman to practical applications in medicine and electronics [14,15,16]. This progress intensified the need for effective regulatory frameworks. Comparing these developmental paths helped the field to understand how societies could build governance systems for new technologies and anticipate future challenges [17,18]. Such comparisons also helped to deal with future regulatory challenges, including international harmonization and transparent risk communication. They also emphasized the importance of developing mechanisms to assess emerging classes of nanoparticles that may not fit existing categories.

This study presented a comprehensive meta-analysis of nanoparticle regulation research to offer insights into this evolving field. The proliferation of nanotechnology boosted the widespread applications, but the unique physicochemical properties that enable innovation also raised concerns regarding human and environmental safety. Traditional regulatory approaches often failed to address nanoparticles’ distinctive characteristics, creating a need for adaptive regulatory paradigms. Therefore, the expansion of suitable frameworks for the safe production, use, and disposal of nanoparticles became a key concern for scientists, industry stakeholders, and policymakers. Eventually, such frameworks would be significant in terms of mitigating risk, besides building public trust and responsible growth of nanotechnology. By combining large-scale text mining with historical comparison, this study aimed to move beyond descriptive trends and provided insight into how regulatory thinking developed, where it currently failed short, and which areas required the focused attention going forward.

## 2. Materials and Methods

### 2.1. Search Keywords

The selected keywords from two categories were on nanoparticles and regulation. “Nanoparticle” (singular) to target particles in the 1–100 nm range [19,20,21,22] and “regulation” were used to specify legal frameworks [19,20,21]. For comprehensive coverage, “nanotechnology” and “nanomaterials” were added, which often appeared interchangeably in policy documents. “Nanomaterials” became the standard term in legislative contexts [22,23,24]. “Nanotechnology” encompassed the broader field of nanoscale manipulation and frequently appeared in policy discussions about wider technological implications [25,26]. The search of 15 combinations by pairing three nano terms with five regulatory keywords (regulation, legislation, policy, safety, and guidelines) were created. Common literature pairings included “nanomaterials legislation” [26,27], “nanotechnology policy” [26], “nanoparticles safety” [24,27], and “nanoparticles guidelines” [26].

### 2.2. Data Source and Preprocess

Our PubMed search covered January 2015 to October 2025, capturing recent regulatory developments. This search yielded 9095 distinct entries. Data extraction covered titles, abstracts, authorship, and metadata. Duplicates, retracted articles, and non-empirical entries (editorials and commentaries) without structured abstracts were removed. Using Python’s Natural Language Toolkit (NLTK) version 3.8.1, stop words, numbers, punctuation, and generic structural terms (“background”, “methods”, and “results”) that reduce topic coherence were removed. Only alphabetic tokens containing three or more characters were included.

### 2.3. Text Mining and Topic Modeling

Computational text mining systematically extracted the semantic patterns from the literature, a necessary approach given the data volume [28,29]. Topic modeling identified thematic structures and their relationships [30]. Topic coherence metrics assessed keyword similarity within topics to ensure semantic validity [30]. Non-negative Matrix Factorization (NMF) with Term Frequency–Inverse Document Frequency (TF-IDF) weighting over Latent Dirichlet Allocation (LDA) were chosen. Comparative studies showed NMF produced better coherent and interpretable results for short scientific abstracts [31,32,33]. NMF splited the document term matrix into two non-negative matrices (topic weights per document and term weights per topic), creating an additive representation that well suited nanoscience terminology.

Titles and abstracts were merged into single-text fields, then applied lowercasing, stop word removal, and lemmatization. The corpus was converted to a document–term matrix using TF-IDF weighting. Frequency filters (min_df = 5, max_df = 0.5) removed rare artifacts and overly common terms that masked distinct topics. Models were tested from K = 1 to K = 12 topics, using UMass coherence as the primary metric [34,35], while prioritizing qualitative interpretability to ensure meaningful research domained.

### 2.4. Keyword Analysis and Word Cloud

Beyond topic modeling, keyword frequency analysis identified the dominant regulatory vocabulary. Frequencies of the top 50 substantive terms were counted to outline our research priorities. A word cloud visualized this distribution with font size and position reflecting term frequency [36]. Though this method could not capture complex semantic relationships or syntax, it offered accessibility advantages. The visualization provided researchers with an immediate overview of prominent themes, complementing the quantitative NMF findings [37].

## 3. Results

### 3.1. Determination of the Optimal Topic Number

NMF topic models with the number of topics (K) ranging from 1 to 12 (Table 1) were evaluated.

Although K = 2 showed the highest UMass coherence score (Table 1), coherence alone did not guarantee human interpretability or sufficient analytical granularity [38]. Models with K between 5 and 7 were examined, as this range better captured major nanoscience research themes. Within this range, both UMass coherence and topic diversity (the proportion of unique words among the top 10–20 terms per topic) were considered. K = 6 achieved the best balance between statistical coherence and diversity. Therefore, K = 6 were selected as the final model for substantive interpretation.

### 3.2. Publication Trends in Nanoparticles Regulation (2015–2025)

The publication trends in nanoparticle regulation from 2015 to 2025 revealed a steady growth throughout the period (Supplementary Figure S1). Publications rose from 112 in 2015 to 309 in 2016, then continued increasing to 495 in 2017. Despite a minor decrease in 2018, the overall trend remained positive. Growth resumed in 2019 (569) and 2020 (670). After 2020, the growth accelerated with 852 publications in 2021, 992 in 2022, and 1068 in 2023. This expansion reflected the growing scholarly attention to regulatory, ethical, and safety aspects of nanoparticles. Publications surged to 1458 in 2024 and 2065 in 2025. This rapid increase emerged from the expanded industrial applications, heightened public and governmental safety concerns, and strengthened global regulatory efforts. Notably, the 2025 data covered up to October, suggesting even higher annual output and confirming the accelerating trend.

### 3.3. Journal Landscape and Disciplinary Distribution

The top journals in nanoparticle regulation research emphasized the field’s interdisciplinary nature (Supplementary Table S1). Outstanding sources included “ACS Applied Materials & Interfaces” (414), “International Journal of Nanomedicine” (259), and “International Journal of Biological Macromolecules” (256), indicating that regulatory discussions originated primarily in applied nanotechnology, biomedicine, and materials science. Publications in “Nanomaterials”, “Journal of Nanobiotechnology”, and “Journal of Colloid and Interface Science” proposed that regulatory concerns arose directly from experimental and toxicological research, rather than policy journals. This pattern suggested that regulatory and safety considerations frequently appeared within applied nanotechnology research, rather than being confined to policy-focused journals. Contributions from “Talanta”, “Advanced Materials”, and “The Science of the Total Environment” emphasized that toxicity, environmental interactions, and analytical methods were fundamental to nanoparticle governance. The journal distribution revealed that regulations were treated as essential elements of scientific research across material, biological, pharmaceutical, and environmental fields, not as a separate concern.

### 3.4. Keyword Frequency

The top 50 keywords, offering a lexical overview of nanoparticle regulation research, were provided in Supplementary Table S2. The most frequent terms: “NPs” (nanoparticles; 8183), “cells” (7101), “potential” (5535), “cell” (5083), and “effects” (4381), emphasized biological interactions, cellular responses, and functional outcomes. Biomedical terms, including “cancer”, “tumor”, “drug”, “delivery”, “treatment”, and “therapy”, appeared frequently, indicating the importance of nanomedicine and therapeutic applications in regulatory discussions. Experimental terms, like “in vivo”, “in vitro”, “expression”, and “release”, showed that regulatory work was closely integrated with laboratory, toxicological, and mechanistic studies.

Safety and materials characterization concepts featured prominently throughout the corpus. Terms, including “toxicity” (3039), “surface” (3041), “exposure” (1938), “protein” (1914), and “stress” (1763), reflected ongoing attention to biological compatibility, toxicological pathways, and nano–bio interface behavior. Material-specific terms showed that the regulation directly connected to the development, assessment, and optimization of specific nanomaterial types. The keywords revealed a field where biological evaluation, therapeutic design, material engineering, and exposure analysis formed the core of nanoparticle regulation research.

### 3.5. Interpretation of Topics

There were six research themes identified through NMF topic modeling (Table 2). TF-IDF weighting helped distinguish semantically distinct domains within the corpus. Topics ranged from “electrochemical energy storage” and “Surface-Enhanced Raman Spectroscopy (SERS) detection” to “metal oxide toxicity”, “antibacterial silver”, “cancer nanotherapy”, and “mRNA delivery”. This breadth confirmed that the field now encompassed engineering, biological interaction, and safety assessment, not just material synthesis. Importantly, these topics showed that regulatory issues were rarely discussed on their own. Instead, they were usually part of application-focused research, where safety, exposure, and performance were considered together. This indicated that regulatory knowledges in nanotechnology were built through experimental and translational studies and not just through regulatory publications.

The first two topics captured technological foundations (Table 2). Topic 1 covered electrochemical nanomaterials, focusing on lithium-based storage and catalytic structures essential for energy applications. Topic 2 shifted to analytical capability, covering research on gold and silver architectures used in SERS-based sensing for food and environmental monitoring. Topics 3 and 4 addressed safety and environmental impacts directly. The model distinguishes between metal oxide toxicity (ZnO and TiO_2_) and antibacterial/green synthesis applications of silver nanoparticles. This separation revealed dual research priorities: advancing performance (Topics 1 and 2), while addressing biological exposure risks (Topics 3 and 4). Topics 5 and 6 represented biomedical applications. Topic 5 covered cancer nanotherapy, particularly photothermal modalities and tumor-targeting strategies. Topic 6 documented the rapid expansion of drug and mRNA delivery systems, which were grown significantly since COVID-19. These topics were interconnected: toxicological findings from Topics 3 and 4 inform the development of safer therapeutic carriers in Topics 5 and 6.

Although Topics 1 and 2 mainly focused on performance-driven applications, such as energy storage and sensing technologies, they also raised important regulatory questions. Issues related to material durability, lifecycle management, and potential environmental release during manufacturing and disposal were often considered only indirectly, indicating a gap between rapid technological development and formal regulatory guidance. The separation between metal oxide toxicity (Topic 3) and antibacterial silver nanoparticles (Topic 4) pointed to different regulatory challenges. Metal oxides were frequently assessed using standardized toxicity and exposure models, whereas silver nanoparticles were usually evaluated in specific application contexts, such as antimicrobial performance. This difference suggested that regulatory approaches may benefit from considering how nanomaterials were used, rather than relying only on material-based classification. The topics related to cancer nanotherapy and drug or mRNA delivery reflected the growing regulatory complexity of advanced biomedical applications. These systems often combined new materials, biological targeting approaches, and therapeutic components, which made it difficult to evaluate using traditional chemical safety frameworks. Their increasing presence suggested a need for regulatory models that could better integrate toxicology, clinical performance, and post-market monitoring.

Overall, the topic structure suggested that nanoparticle regulation was developing alongside scientific innovation, but not always at the same pace. Regulatory understanding was often shaped within scientific research, while formal governance frameworks may take longer to adjust, particularly for complex and rapidly evolving applications.

### 3.6. Word Cloud

**Figure 1:**
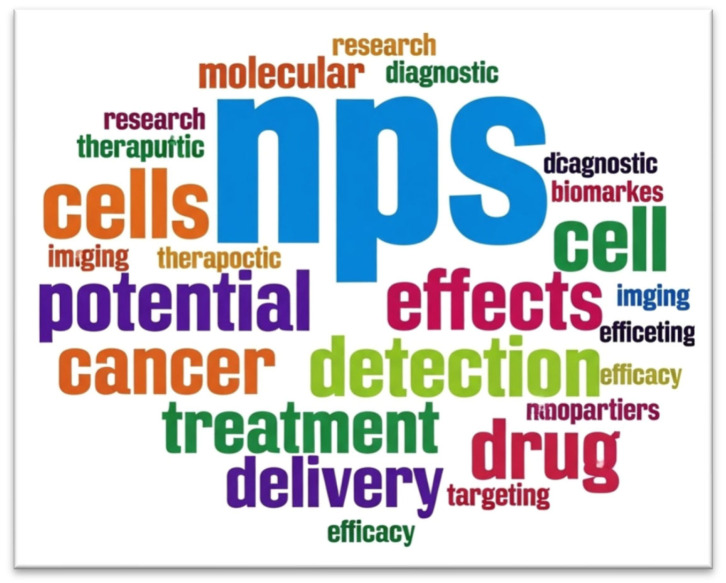
The word cloud visualization of the most frequently occurring keywords extracted from titles and abstracts of nanoparticle regulation-related publications (2015–2025). The size of each word reflected its relative frequency within the dataset after text preprocessing, including stop word removal and lemmatization. Prominent terms indicated the strong emphasis on biological interactions, therapeutic applications, toxicity, and exposure assessment in nanoparticle research.

A word cloud visualization corresponding to keyword frequencies was provided in Figure 1. “NPs” dominated the visualization, as expected. The surrounding terms revealed more: “cells”, “cancer”, “treatment”, and “drug” appeared prominently, showing that biomedical applications drive regulatory discourse more than material synthesis alone. The vocabulary emphasized the function over structure. Application terms, like “delivery” and “therapeutic”, sat alongside mechanistic descriptors, including “effects”, “toxicity”, and “in vivo”. Nanoparticles were consistently discussed in the context of their biological interactions, not in isolation. Diagnostic applications were also featured significantly. The recurrence of “detection”, “biomarkers”, and “molecular” reflected the integration of nanotechnology into sensing platforms. The visualization confirmed a field, where material engineering connected directly to therapeutic and diagnostic applications. This visualization complemented the quantitative keyword frequency analysis, presented in Supplementary Table S2.

These results mainly exhibited how regulatory issues naturally arose within scientific research, rather than being addressed separately in dedicated governance literature.

## 4. Discussion

### 4.1. Evolution of Nanoparticle Regulation and Historical Parallels

Nanoparticle regulations from 2015 to 2025 shared several characteristics with the historical development of radiation governance. Radiation regulation evolved from scientific discovery through commercial deployment to structured oversight following critical incidents and evidence of harm [39,40,41,42]. Table 3 provided a comparative framework for understanding societal responses to emerging technologies with uncertain, but potentially significant risks. Nanotechnology’s trajectory (Table 4) showed similar expansion from conceptual foundations to diverse industrial and biomedical applications. Our bibliometric analysis revealed that regulatory attention increased sharply after 2019 with a sustained and accelerating increases in publication output, rather than exponential growth in publications (Supplementary Figure S1). This trend aligned with major policy changes, such as the European Chemicals Agency’s (ECHA’s) updated nanoform registration guidance [39,43]. While radiation regulation developed reactively after catastrophic events, nanoparticle governance benefited from existing regulatory infrastructures that enabled earlier institutional engagement and anticipatory risk management [39,44,45]. The accelerated regulatory discourse reflected both nanotechnology’s proliferation and lessons learned from previous regulatory experience.

### 4.2. Thematic Structure and Knowledge Evolution

The six identified topics showed that nanotechnology research branched into specialized, yet connected domains across engineering, toxicology, life sciences, and medicine. Energy storage and sensor technologies expanded with industrial and analytical demands, while metal oxide toxicity and antibacterial silver research provided insights into exposure pathways, oxidative stress, and biological reactivity. Cancer nanotherapy and mRNA/lipid nanoparticle delivery systems reflected nanotechnology’s growing role in advanced biomedical applications. The TF-IDF-based NMF approach extracted distinct and interpretable topics, confirming structural coherence across the six domains. These topics existed, not as isolated fields, but as a network of mutually reinforcing research directions that collectively advanced technical efficiency, biological safety, and medical applicability.

The topics evolved interdependently, forming networks that connected technical efficiency, biological safety, and medical application. Toxicity studies (Topic 3) informed safety evaluations of drug or gene delivery platforms (Topic 6). Antibacterial silver nanoparticle (AgNP) research (Topic 4) advanced the understanding of cellular uptake and reactivity, influencing therapeutic nanoparticle design. This interconnected structure resembled radiation regulation’s evolution from discovery through risk identification to assessment frameworks [39,46], but it proceeded faster and in multiple directions.

### 4.3. Interdisciplinary Integration in Regulatory Development

The journal distribution (Supplementary Table S1) revealed the interdisciplinary nature of nanoparticle regulation research with contributions from materials science, toxicology, nanomedicine, and environmental chemistry. This publication trend showed that regulatory thinking was embedded within scientific research rather than emerging only from policy fields. The increase in toxicity, exposure, and nano–bio interaction research indicated that scientific communities across disciplines now incorporated regulatory considerations into their research.

This integrated trajectory differed from the historical pattern, seen in many earlier emerging technologies, where regulation typically lagged behind innovation [47]. In nanotechnology, regulatory frameworks developed alongside scientific advances. The 2019 ECHA nanoform registration revision [39] included detailed guidance for the characterization, reporting, and substance grouping for nanomaterials. The alignment between regulatory updates and increased scholarly output indicated co-evolution, where standards, testing methods, and scientific practices developed together rather than sequentially.

Interdisciplinarity created coordination challenges, despite its benefits. Each field contributing to nanoparticle research operated with distinct methodological frameworks, exposure assumptions, and risk assessment priorities. The breadth of this ecosystem complicated efforts to harmonize regulatory approaches across sectors. As radiation governance demonstrated, effective oversight required alignment among scientific, industrial, and regulatory actors [48,49]. Nanoparticle governance appeared to be entering this phase of negotiated integration, where cross-sector collaboration would be essential for establishing robust, flexible, and widely applicable regulatory standards.

### 4.4. Emerging Research Patterns and Regulatory Implications

Our results presented key considerations for future nanoparticle governance. The focus on applications suggested that regulation may need to be tailored and use specific frameworks. Environmental risk assessment became highly important through studies on toxicity and oxidative stress, much like how radiation protection slowly expanded to include environmental and workplace safety standards [50,51,52,53]. Industrial development, biomedical innovation, and environmental monitoring coexisted in the research landscape, indicating that effective regulation required knowledge exchanges between research and regulatory institutions. The overlap observed between regulation and application topics indicated that this interaction was already occurring, supporting better agile and informed policy development.

### 4.5. Methodological Advances in Regulatory Research

Text mining, frequency analysis, and topic modeling provided insights that were difficult to obtain through traditional reviews, creating a systematic framework for mapping regulatory issues. In particular, the TF-IDF-based NMF topic modeling approach yielded clearer and better coherent thematic separation than probabilistic methods, allowing the identification of regulatory patterns embedded within experimental, toxicological, and biomedical research. Coherence-validated topics revealed how regulatory considerations span multiple scientific domains, while word cloud visualization displayed dominant biological and therapeutic vocabulary. These computational methods, combined with knowledge structuring principles in innovative research, enabled data-driven regulatory assessment by revealing connections between scientific activity and governance needs [54,55].

### 4.6. Challenges and Future Directions

Dataset limitations restricted our ability to capture early debates and regional regulatory approaches, particularly in countries where non-English scholarship and policy were expanding rapidly. Since nanotechnology evolved faster than many historical technologies, regulatory frameworks would be needed greater adaptability to incorporate new scientific knowledge, while maintaining safety protocols.

One of the main challenges for nanoparticle regulation was the wide diversity of materials and applications. Nanoparticles differed not only in size and composition, but also in surface properties, coatings, and functional use. As a result, regulatory frameworks based solely on chemical identity or mass-based thresholds were often insufficient to capture relevant exposure and risk profiles. Another challenge was the gap between rapid scientific innovation and the slower pace of regulatory adaptation. As shown by the growth in advanced biomedical and multifunctional nanoparticles in this analysis, new materials often entered research and early-stage applications before standardized testing guidelines or clear regulatory pathways were established. This created uncertainty for researchers, industry, and regulators alike. Lessons from radiation governance suggested that effective regulation developed through iterative refinement rather than fixed rules. Radiation standards evolved as scientific understanding improved, exposure pathways became clearer, and international coordination strengthened. A similar adaptive approach may be necessary for nanotechnology, where risk assessment frameworks must evolve alongside emerging evidence rather than relying on static classifications.

Future research should include longitudinal analyses of regulatory strategy performance, comparative evaluations of regional governance models to identify different regulatory strengths and weaknesses, and studies on balancing international harmonization with local policy needs. Future regulatory strategies may benefit from shifting toward use-specific and exposure-driven assessment models. Instead of treating all nanoparticles within a single category, regulatory evaluation could focus on how and where materials were used, the likelihood of human or environmental exposure, and the persistence of nanoparticles across their life cycle. This approach aligns closely with the application-driven nature of current identified nanotechnology research in this study. The increasing volume and complexity of nanotechnology data also indicated a growing role for data-driven tools in regulatory science. Text mining, machine learning, and computational modeling may assist in grouping similar nanomaterials, predicting potential hazards, and identifying priority areas for experimental testing. Such tools were not intended to replace regulatory judgment, but rather to support evidence-based decision-making in a rapidly expanding field. The complexity of nanoparticle interactions and application diversity suggested that artificial intelligence (AI) and machine learning would play larger roles in regulatory assessment. These tools could enhance risk prediction and material classification by processing high-dimensional datasets, like those in our text mining and topic modeling analyses. Developing agile, data-informed systems that responded to rapid innovation would be essential for safe nanotechnology advancement across industrial, medical, and environmental applications.

Finally, international coordination remained essential. Nanoparticle research, manufacturing, and commercialization operated across borders, yet regulatory requirements often differed by region. Greater alignment among international guidelines and data-sharing practices could reduce the duplication of testing efforts, while maintaining safety standards. Experiences from radiation protection demonstrated that harmonized frameworks could coexist with national regulatory systems, when supported by shared scientific principles.

Together, these considerations suggested that future nanoparticle governance should emphasize flexibility, transparency, and continuous learning, ensuring that regulatory systems could respond effectively to ongoing scientific and technological change.

## 5. Conclusions

This study presented that nanoparticle regulation was maturing rapidly, shaped by interdisciplinary advances and historical precedents, like radiation governance, yet requiring flexible approaches due to nanotechnology’s accelerated development. Materials science, toxicology, biomedical research, and environmental studies converged across publication trends, journal distributions, topics, and lexical patterns, showing that scientific inquiry and regulatory thinking would advance together. Computational tools, including text mining, frequency analysis, and TF-IDF-based NMF topic modeling, provided systematic insights into emerging regulatory challenges. As academic, industrial, and regulatory collaboration grew alongside international coordination, nanoparticle regulation increasingly supported innovation, while protecting human and environmental health. Maintaining adaptable, equitable, and globally coherent governance became crucial for responsible nanotechnology expansion.

## Figures and Tables

**Table 1:** Comparison of evaluation metrics for NMF models across different numbers of topics (K).

K	Coherence (UMass)	Topic Diversity	Unique Top Words (Ratio)
1	−1.36	1	20/20 (1.00)
2	−1.32	0.98	39/40 (0.98)
3	−1.38	0.93	56/60 (0.93)
4	−1.49	0.89	71/80 (0.89)
5	−1.56	0.85	85/100 (0.85)
**6**	**−1.63**	**0.84**	**101/120 (0.84)**
7	−1.68	0.86	120/140 (0.86)
8	−1.66	0.85	136/160 (0.85)
9	−1.74	0.85	153/180 (0.85)
10	−1.75	0.86	171/200 (0.86)
11	−1.82	0.84	185/220 (0.84)
12	−1.86	0.81	194/240 (0.81)

Note: UMass indicates UMass coherence score (higher was better; in this case, closer to 0). The bold row (K = 6) represents the selected model for this study. Values were rounded to two decimal places.

**Table 2:** Summary of six topics derived from topic modeling.

Topic	Topic Title	Top Keywords
1	Energy Storage and Electrochemical Nanomaterials	Metal, Carbon, Performance, Energy, Batteries, Lithium, Density, Nanoparticles, Ion, Structure, Capacity, Pt, Catalytic, Composite, Temperature
2	SERS-Based Nanoparticle Sensors and Detection Technologies	Detection, SERS, Food, Au, Sensor, AuNPs, Sensitive, Gold, Raman, Rapid, Ag, Sensitivity, Signal, Surface, Sensing
3	Metal Oxide Nanoparticle Toxicology and Oxidative Stress	NPs, ZnO, TiO_2_, Ag, Oxide, Zinc, Toxicity, Mg, Exposure, Stress, CuO, Nanoparticles, Plant, Oxidative, PLGA
4	Silver Nanoparticles for Antibacterial Applications and Green Synthesis	AgNPs, Silver, Antibacterial, Ag, Antimicrobial, AgNP, Nanoparticles, Extract, Green, Activity, Synthesis, Synthesized, Bacterial, Cells, Effects
5	Cancer Nanotherapy and Photothermal/Drug-Based Treatments	Tumor, Cancer, Therapy, Dox, Breast, Treatment, Targeting, Drug, Photothermal, Targeted, Therapeutic, Antitumor, Tumors, Imaging, Microenvironment
6	Nanoparticle-Based Drug and mRNA Delivery Systems	Delivery, Drug, Nanoparticles, Lipid, mRNA, Release, Inflammatory, Loaded, Potential, in Vitro, in Vivo, Treatment, Safety, Mice, Toxicity

Abbreviations. Ag: silver, Au: gold, CuO: cupric oxide, Mg: magnesium; NPs: nanoparticles, PLGA: poly(lactic-co-glycolic acid), Pt: platinum, SERS: Surface-Enhanced Raman Scattering, TiO_2_: titanium dioxide, and ZnO: zinc oxide.

**Table 3:** General overview of radiation regulations.

Phase	Time Period	Key Developments
Early Discovery	Late 19th– Early 20th Century	Discovery of radioactivity by Henri Becquerel (1896) Isolation of polonium and radium by Marie and Pierre Curie (1898) Introduction of the term ‘radioactivity’ by Marie Curie
Scientific and Commercial Advancement	1900s–1950s	Medical application of X-rays (early 1900s) Discovery of nuclear fission (1930s) Development of atomic weapons (1940s) Commercialization of nuclear power plants (1950s)
Major Radiation Incidents	1945–2011	Atomic bombings of Hiroshima and Nagasaki (1945) Three Mile Island accident (1979) Chernobyl disaster (1986) Fukushima Daiichi nuclear disaster (2011)
Establishment of Regulatory Bodies	1928–Present	ICRP (1928) IAEA (1957) NRC (1975) Various national regulatory bodies (e.g., NSSC in South Korea, 2011; NRA in Japan, 2012)
Regulatory Framework Development	Ongoing	Setting radiation exposure limits Establishing safety standards for nuclear facilities Regulations for handling and transporting radioactive materials Guidelines for radioactive waste management
International Cooperation	Ongoing	Implementation of international standards (IAEA, ICRP) Regional standardization (e.g., EAEC) International agreements (e.g., NTBT)

Abbreviations: EAEC: European Atomic Energy Community, IAEA: International Atomic Energy Agency, ICRP: International Commission on Radiological Protection, NSSC: Nuclear Safety and Security Commission, NRA: Nuclear Regulation Authority, NRC: Nuclear Regulatory Commission, and NTBT: Nuclear Test Ban Treaty.

**Table 4:** General aspects of nanoparticle research and regulations.

Phase	Time Period	Key Developments
Conceptual Foundations	1959–1980s	Richard Feynman’s lecture “There’s Plenty of Room at the Bottom” (1959) Coining of the term “nanotechnology” by Norio Taniguchi (1974) Invention of Scanning Tunneling Microscope (STM, 1981)
Fundamental Discoveries	1980s–1990s	Discovery of fullerenes (C60, 1985) Discovery of carbon nanotubes (1991)
Commercial Applications	1990s–Present	Cosmetics and sunscreens (1990s) Electronics and medical devices (2000s) Advanced materials and energy storage (2010s)
Emerging Research Fields	Ongoing	Medicine: drug delivery, bioimaging, and cancer treatment Electronics: Nano-transistors and nanowires Materials Science: coatings, composites, and high-performance products
Regulatory Concerns	Early 2000s–Present	Safety of nanomaterials Inadequacy of existing assessment methods Environmental impact Bioaccumulation potential
Establishment of Regulatory Bodies	2000s–Present	US EPA guidelines ECHA regulations REACH standards
Key Regulatory Measures	Ongoing	Definition and classification of nanomaterials Product labeling requirements Safety assessment guidelines Ethical guidelines for research and development
International Coordination	Ongoing	OECD and ISO guidelines EU, US, and Japan regulatory systems Developing countries were establishing regulations based on developed countries’ models

Abbreviations. ECHA: European Chemicals Agency; EPA: Environmental Protection Agency; EU: European Union; ISO: International Organization for Standardization; OECD: Organization for Economic Co-operation and Development; REACH: Registration, Evaluation, Authorization, and Restriction of Chemicals; and US: United States.

## Data Availability

All are contained in the manuscript. Additional figures/tables were contained in the Supplementary Information.

## Supplementary Materials

The following supporting information can be downloaded at https://www.mdpi.com/article/10.3390/nano16020134/s1: Figure S1: Annual number of PubMed-indexed publications related to nanoparticle regulation from January 2015 to October 2025. Publications were identified using predefined combinations of nanoparticle-related and regulatory keywords. The figure illustrates a steady increase in research output over the study period with a marked acceleration after 2019. Data for 2025 include publications indexed up to October 2025. Table S1: Top Twenty-five Journals Ranked by Publication Count. Table S2: Top Fifty Tokens by Frequency.
