# Supplementary material for: The Evolution of Nanoparticle Regulation: A Meta-Analysis of Research Trends and Historical Parallels (2015–2025)
Source: Nanomaterials (Basel). 2026 Jan 19;16(2):134. doi: 10.3390/nano16020134 (PMC12844885; doi:10.3390/nano16020134)
Supplement: Supplementary file 1 [file nanomaterials-16-00134-s001.zip › nanomaterials-4075297-supplementary.pdf]

# Supplementary Information

## The Evolution of Nanoparticle Regulation: A Meta-Analysis of Research Trends and Historical Parallels (2015-2025)

**Sung-Kwang Shin[1†], Niti Sharma[2†], Seong Soo A. An[2*] and Meyoung-Kon (Jerry) Kim[3*]**

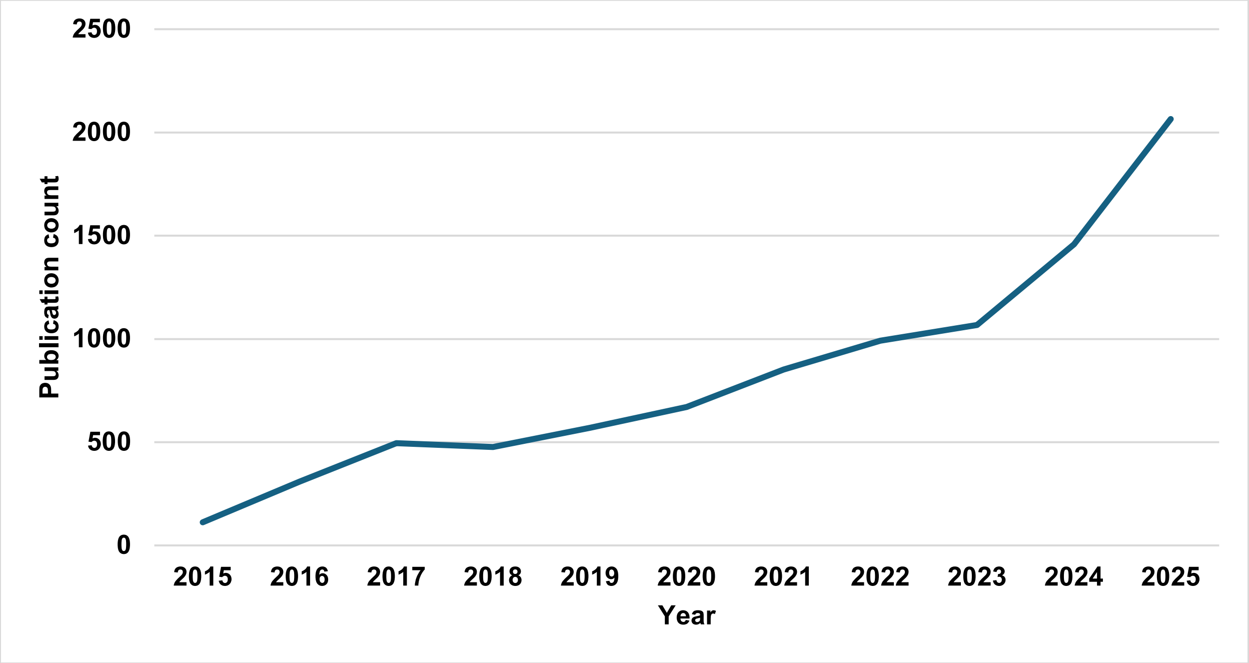


**Supplementary Figure S1.** Annual number of PubMed-indexed publications related to nanoparticle regulation from January 2015 to October 2025. Publications were identified using predefined combinations of nanoparticle-related and regulatory keywords. The figure illustrates a steady increase in research output over the study period, with a marked acceleration after 2019. Data for 2025 include publications indexed up to October 2025.

**Supplementary Table S1.** Top Twenty-five Journals Ranked by Publication Count.

| Rank | Journal Title | Publication Count |
|---|---|---|
| 1 | ACS Applied Materials & Interfaces | 414 |
| 2 | International journal of nanomedicine | 259 |
| 3 | International Journal of Biological Macromolecules | 256 |
| 4 | Nanomaterials | 231 |
| 5 | Journal of Nanobiotechnology | 172 |
| 6 | Journal of Colloid and Interface Science | 166 |
| 7 | Nanoscale | 154 |
| 8 | International Journal of Pharmaceutics | 153 |
| 9 | Pharmaceutics | 149 |
| 10 | Talanta | 141 |
| 11 | Food Chemistry | 133 |
| 12 | Scientific Reports | 131 |
| 13 | RSC Advances | 129 |
| 14 | International Journal of Molecular Sciences | 123 |
| 15 | Advanced Materials | 115 |
| 16 | Analytica Chimica Acta | 110 |
| 17 | Small | 108 |
| 18 | Molecules | 108 |
| 19 | The Science of the Total Environment | 101 |
| 20 | Colloids and Surfaces. B, Biointerfaces | 99 |
| 21 | Biosensors & Bioelectronics | 92 |
| 22 | Chemosphere | 92 |
| 23 | ACS Omega | 88 |
| 24 | Advanced Science | 88 |
| 25 | Journal of Controlled Release | 74 |

**Supplementary Table S2**. Top Fifty Tokens by Frequency.

| Rank | Token | Frequency | Rank | Token | Frequency |
|---|---|---|---|---|---|
| 1 | nps | 8183 | 26 | human | 2278 |
| 2 | cells | 7101 | 27 | different | 2189 |
| 3 | potential | 5535 | 28 | promising | 2172 |
| 4 | cell | 5083 | 29 | oxide | 2138 |
| 5 | effects | 4381 | 30 | development | 2066 |
| 6 | cancer | 4375 | 31 | model | 2037 |
| 7 | detection | 4236 | 32 | growth | 2027 |
| 8 | treatment | 4141 | 33 | efficiency | 2020 |
| 9 | delivery | 4037 | 34 | stability | 2020 |
| 10 | drug | 3864 | 35 | agnps | 2017 |
| 11 | activity | 3705 | 36 | strategy | 2000 |
| 12 | effect | 3311 | 37 | acid | 1984 |
| 13 | tumor | 3255 | 38 | silver | 1972 |
| 14 | in vivo | 3088 | 39 | mice | 1968 |
| 15 | surface | 3041 | 40 | exposure | 1938 |
| 16 | toxicity | 3039 | 41 | protein | 1914 |
| 17 | regulation | 2939 | 42 | nanoparticle | 1868 |
| 18 | in vitro | 2922 | 43 | control | 1825 |
| 19 | expression | 2620 | 44 | magnetic | 1789 |
| 20 | release | 2614 | 45 | levels | 1776 |
| 21 | therapeutic | 2598 | 46 | stress | 1763 |
| 22 | food | 2588 | 47 | gene | 1741 |
| 23 | efficacy | 2572 | 48 | gold | 1682 |
| 24 | therapy | 2547 | 49 | clinical | 1622 |
| 25 | enhanced | 2293 | 50 | response | 1620 |
